# Serum Uric Acid as a Marker of Coronary Calcification in Patients with Asymptomatic Coronary Artery Disease with Preserved Left Ventricular Pump Function

**DOI:** 10.1155/2013/129369

**Published:** 2013-03-31

**Authors:** A. E. Berezin, A. A. Kremzer

**Affiliations:** ^1^Internal Medicine Department, State Medical University, Zaporozhye 69035, Ukraine; ^2^Clinical Pharmacology Department, State Medical University, Zaporozhye 69035, Ukraine

## Abstract

*Objective*. To evaluate the interrelation between serum uric acid and artery calcification in asymptomatic coronary artery disease subjects. *Design and Methods*. 126 subjects with previously documented asymptomatic coronary artery disease were enrolled in the study. *Results*. Mean value of serum uric acid level was 23.84 mmol/L (95% confidence interval (CI)  =  15.75–31.25 mmol/L). In multivariate Cox regression analysis, the results showed that serum uric acid levels (odds ratio (OR) = 1.42, 95% CI = 1.20–1.82; *P* < 0.001), osteopontin (OR = 1.14, 95% CI = 1.12–1.25; *P* < 0.001), osteoprotegerin (OR = 1.45, 95% CI  =  1.20–1.89; *P* < 0.001), type 2 diabetes mellitus (OR = 1.41, 95% CI  =  1.20–1.72; *P* < 0.001), and total cholesterol (OR = 1.13, 95% CI = 1.10–1.22; *P* < 0.001) were factors that independently associated with coronary artery calcification. The Cox models suggested that high quartile of serum uric acid level is very significant in predicting Agatston score index. In conclusion, we suggested that high quartile of serum uric acid level (cutoff point equaled 35.9 mmol/L) was a very significant predictor of coronary calcification examined by Agatston score index in subjects with asymptomatic coronary artery disease.

## 1. Background

Hyperuricemia is frequently present in patients with symptomatic heart failure, acute coronary syndromes, arterial hypertension, and atrial fibrillation and in patients with type 2 diabetes mellitus [1–3]. Current evidence suggests that serum uric acid could be a marker of oxidative damage [4]. Serum uric acid is also considered a useful biomarker for mortality and an indicator of a poor prognosis in high-risk patients with several cardiovascular diseases [5–7]. Recently clinical studies have shown that serum uric acid inversely correlates with left ventricular ejection fraction, serum creatinine, and blood urea nitrogen in patients with heart failure [1]. There is a significant association between serum uric acid and circulating levels of proinflammatory cytokines among subjects with chronic heart failure [8]. Serum uric acid is often discussed as a risk factor for acute kidney injury, which adversely affects renal blood flow autoregulation, glomerular filtration rate, and promotes inflammation and angiogenesis [9]. However, the principal mechanism that contributes to biological effects of serum uric acid in patients with asymptomatic coronary artery disease without reducing left ventricular pump function is still to be understood. It has been postulated that serum uric acid plays a pivotal role in the pathogenesis of cardiovascular diseases affecting xanthine oxidase pathway that contributes to the production of active oxygen species generation with deterioration of cells membranes [10]. Reactive oxygen species contribute to vascular oxidative stress and endothelial dysfunction, which are associated with the risk of atherosclerosis, damages of both cardiomyocytes and vascular endothelium inducing disturbances of myocardial contractility and vasoconstriction also [11]. Indeed, few studies have investigated the association of hyperuricemia with subclinical heart failure and atherosclerosis [12–15]. However, we do not know exactly whether serum uric acid is only a marker rather than a causal factor causing coronary calcification in asymptomatic ischemic heart disease [8, 10].

*The aim* of the study was to evaluate the interrelation between serum uric acid and coronary artery calcification in asymptomatic coronary artery disease subjects with preserved left ventricular systolic function.

## 2. Design and Methods

The population of the study was structured retrospectively after determination of coronary artery disease by contrast-enhanced spiral computer tomography angiography in one hundred twenty-six asymptomatic subjects. All subjects gave their written informed consent to participate in the study before enrollment.

*The Included criteria* are asymptomatic coronary artery disease, preserved left ventricular ejection fraction, age more than 18 years, sinus rhythm, and written informed consent for participation in the study.

*Excluded criteria* are symptomatic chronic heart failure, left ventricular ejection fraction (LVEF) ≤40%, uncontrolled diabetes mellitus, severe kidney and liver diseases that have ability to independently influence the clinical outcomes, malignancy, unstable angina, Q-wave and non-Q-wave myocardial infarction within 30 days before study begins, creatinin plasma level above 440 *μ*mol/L, eGFR index <35 mL/min/1.73 m^2^, brain injury within 3 months before enrollment, body mass index above 30 kg/m^2^ and less than 15 kg/m^2^, pulmonary edema, tachyarrhythmia, valvular heart disease, thyrotoxicosis, ischemic stroke, intracranial hemorrhage, acute infections, surgery, trauma, all ischemic events during the previous 3 months, inflammatory conditions within 1 month, and incidence of neoplasm were ruled out by careful medical history and physical examination before the study began, pregnancy, and an implanted pacemaker.

### 2.1. Echocardiography Examination

Echocardiography in B-mode was performed on the Recommendation of American Society of Echocardiography on scanner ACUSON (Siemens, Germany) using a transducer with a frequency of 2.5–5 MHz. End-diastolic and end-systolic LV volumes were obtained using a two-dimensional reference sector according to Simpson's method, and LV ejection fraction (LVEF) was calculated according to conventional methods [16].

### 2.2. Contrast-Enhanced Spiral Computer Tomography Angiography

Coronary vessel wall and plaque's geometrical and compositional parameters were measured on contrast-enhanced spiral computer tomography (CT) angiography [17]. CT was performed on a “Somatom Volume Zoom” scanner (Siemens, Erlangen, Germany) with 2 rows of detectors (32 × 2 CT system) during end-expiratory breath hold. After noncontrast localization image acquisition, an injection of a nonionic contrast “Omnipak” (Amersham Health, Ireland) was used to determine optimal coronary arterial image. Images were reconstructed in 0.6 mm axial slices. Coronary artery calcification was quantified by calculating the Agatston' score index and calcification mass measurement [18]. We determined calcified atherosclerotic plaque, high-density noncalcified plaque (HD-NCP), and low-density noncalcified plaque (LD-NCP). Calcified atherosclerotic plaques (CAP) were classified with attenuation values of 150 HU (Hounsfield units) or greater, as HD-NCP with 30 to 149 HU and as LD-NCP with −100 to +30 HU [19, 20].

### 2.3. Glomerular Filtration Ratio Estimation

Estimated glomerular filtration ratio (eGFR) was calculated by MDRD formula [21].

### 2.4. Blood Sampling

All samples were collected in cooling vacutainer and after that they were immediately centrifuged (4°C for 6.000 × 15 min). After centrifugation serum was coded and stored at −70° in a refrigerator until used.

### 2.5. Serum Uric Acid Measurement

Serum uric acid level was measured by enzymatic methods [22] using chemical analyzer Beckman Synchron LX20. Analytical range average for serum uric acid was 0.5–12 mg/dL.

### 2.6. Osteopontin and Osteoprotegerin Levels Determination

Osteopontin and osteoprotegerin levels were measured by ELISA technique. For both biomarkers examination, Humans Quantikine ELISA Kits (R&G, United Kingdom) were used. All determinations were done by duplicate. The mean intra-assay coefficients of variation were <10% for all cases.

### 2.7. High-Sensitive C-Reactive Protein Level Determination

High-sensitive C-reactive protein (hs-CRP) level was measured by nephelometric technique and obtained with “AU640 Analyzer” (Olympus Diagnostic Systems Group, Japan).

### 2.8. Cholesterol Level Measurements

Concentrations of total cholesterol (TC) and high-density lipoproteins (HDL) cholesterol were determined by Dimension Clinical Chemistry System (Dade Behring Inc., Newark, NJ, USA). The levels of low-density lipoproteins (LDLs) cholesterol were calculated by using the formula of Friedewald W. T., Levy R. I., Fredrickson D. S. (1972).

### 2.9. Statistical Analysis

All statistical analyses were performed in SPSS for Windows v. 20.0 (SPSS Inc., Chicago, Il, USA, 2011). All values were given as mean and 95% confidence interval (CI) or median and percentiles. An independent group *t*-test was used for comparisons of all interval parameters meeting the criteria of normality and homogeneity of variance. For interval parameters not meeting these criteria, the nonparametric Mann-Whitney test was used to make comparisons between variables. Comparisons of categorical variables between groups were performed using the Chi^2^ test and the Fisher exact test. Osteopontin, osteoprotegerin, serum uric acid, and hs-CRP concentrations were normally distributed (using Kolmogorov-Smirnov test), and data was not positively skewed. However, the data was not transformed. The potential factors that may be associated with severity of coronary atherosclerosis and calcification were identified first with the univariant analysis (ANOVA); Cox multivariant analyses were used to identify predicted value of determined factors. Receiver operating characteristic (ROC) curves were configured to establish cutoff points of serum uric acid level that optimally predicted coronary atherosclerosis. A calculated difference of *P* < 0.05 was considered significant. 

## 3. Results

General characteristics of study patients are presented in Table 1. A group of persons, which were surveyed, were predominantly males (58.7%) at the age of 58.34 ± 9.60 years, with mean HbA1c level equal to 6.8%, with hyperlipidemia, mild-to-moderate increase of hs-CRP, and history of hypertension. Type 2 diabetes mellitus was determined in 36.5% cases. Calcified atherosclerotic plaques were determined in 96% cases; HD-NCP and LD-NCP were found in 31% and 25%, respectively. Numerous coronary arteries with plaques determined were 36.5% (1 vessel), 33.3% (2 vessels), and 20.2% (3 and more vessels). All patients were treated according to current clinical guidelines with diet, lifestyle modification, and drug therapy that included angiotensin-converting enzyme inhibitors/angiotensin-2 receptor blockers, aspirin, or other antiaggregants, statins, and metformin if necessary.

Analysis of obtained results has revealed that mean value of serum uric acid level was 23.84 mmol/L (95% CI = 15.75–31.25 mmol/L). Data was distributed in quartiles (Q) as follows: for Q1 mean value of serum uric acid was 12.25 mmol/L (95% CI = 11.31–13.19 mmol/L), for Q2 mean value of serum uric acid was 17.33 mmol/L (95% CI = 16.45–18.22 mmol/L), for Q3 mean value of serum uric acid was 25.29 mmol/L (95% CI = 23.53–27.09 mmol/L), and for Q4 mean value of serum uric acid was 39.27 mmol/L (95% CI = 35.05–43.48 mmol/L). Using the 95th percentile of serum uric acid within normal ranges as 32 mmol/L for male and 36 mmol/L for females, we determined that there are 9 (7.1%) males and 7 (5.6%) females with hyperuricemia in the population observed. We predisposed that low frequencies of hyperuricemia occurring in patient population did not require a contribution depending on sex-adjusted value of serum uric acid concentration for further regression analysis.

Univariant regression analysis suggested that serum uric acid plasma levels were directly related to osteopontin (*r* = 0.536, *P* < 0.001), LD-CAP (*r* = 0.424, *P* < 0.001), Agatston' score index (*r* = 0.372, *P* = 0.016), type two diabetes mellitus (*r* = 0.205, *P* = 0.055), total cholesterol (*r* = 0.326, *P* < 0.001), osteopontin (*r* = 0.246, *P* = 0.027), coronary artery stenosis of more 50% (*r* = 0.286, *P* = 0.012), eGFR (*r* = 0.348, *P* < 0.001), and creatinine plasma level (*r* = 0.321, *P* < 0.001) and inversely correlated to LVEF (*r* = −0.735, *P* < 0.001). At the same time, the significant association between serum uric acid with fasting glucose, HbA1c, mean systolic blood pressure, and premature coronary artery disease in family anamnesis and medications for the entire cohort of the patients was not determined.

Generalized regression linear models for two dependent variables which contribute to coronary calcification included coronary artery stenosis of more 50% and Agatston score index and are shown in Table 2. We revealed that three factors, such as type two diabetes mellitus, total cholesterol, and osteoprotegerin, can be considered to contribute to coronary artery stenosis severity. However, osteoprotegerin became a factor that associates with Agatston score index. After excluding, in both these models, type two diabetes mellitus as a factor, we suggested that osteoprotegerin, osteopontin, and serum uric acid significantly affected the variable of interest (Agatston score index) only (Table 3).

### 3.1. Factors Associated with Coronary Artery Calcification

**Figure 1:**
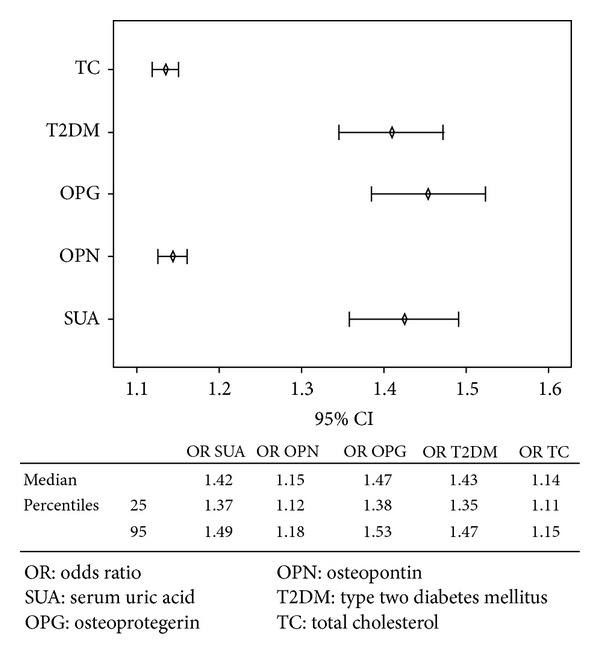
Cox regression analysis shows factors that independently associate with coronary artery calcification appraised by calculation of Agatston score index value.

In multivariate Cox regression analysis, the results showed that serum uric acid (odds ratio (OR) = 1.42, 95% CI = 1.20–1.82, and *P* < 0.001), osteopontin (OR = 1.14, 95% CI = 1.12–1.25, and *P* < 0.001), osteoprotegerin (OR = 1.45, 95% CI = 1.20–1.89, and *P* < 0.001), type two diabetes mellitus (OR = 1.41, 95% CI = 1.20–1.72, and *P* < 0.001), and total cholesterol (OR = 1.13, 95% CI = 1.10–1.22, and *P* < 0.001) were factors that independently associated with coronary artery calcification (Figure 1). After adjustment for significant predictors, including osteopontin, osteoprotegerin, type two diabetes mellitus, total cholesterol, and demographic variables (age, sex) serum uric acid became useful to predict Agatston score index value (HR = 1.12, 95% CI = 1.01–1.52, and *P* < 0.001).

Multivariable-adjusted odds ratios revealed that high quartile of serum uric acid had been considerable in predicting the value for coronary calcification when compared with other quartiles of serum uric acid (Table 4).

### 3.2. Effect of Serum Uric Acid Level on the Sensitivity and Specificity of Predicting Models for Coronary Artery Calcification

**Figure 2:**
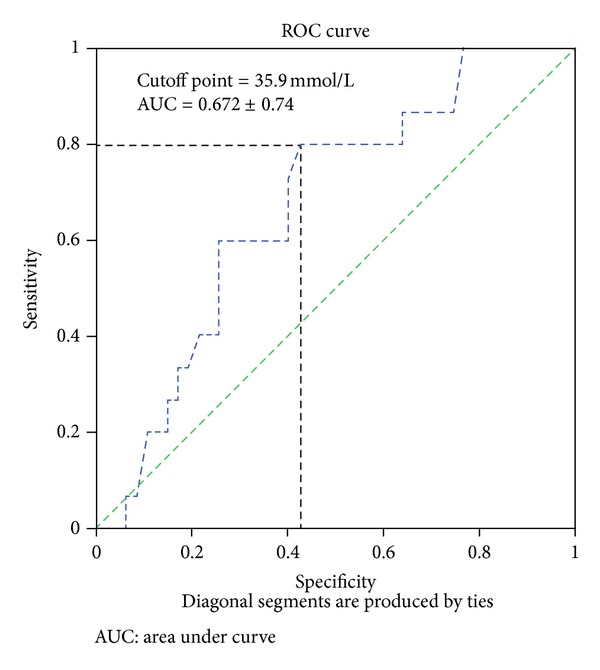
Sensitivity and specificity of the prognostic value of serum uric acid for coronary calcification. Results of ROC curves analysis.

To determine the sensitivity and specificity of the prognostic value of serum uric acid, ROC curve was developed. The Cox models suggested that high quartile serum uric acid level was very significant in predicting Agatston score index (Wald *χ* ^2^ = 147.83, *P* < 0.001). The cutoff point of SUA for this model was 35.9 mmol/L. The sensitivity and specificity of the models were 80.0% and 59.2%, respectively, for predicting coronary artery calcification; area under curve was 0.672 ± 0.74 (Figure 2). When serum uric acid was added to these models as first, second, and third quartiles, it failed to predict Agatston score index (Wald *χ* ^2^ = 14.28, *P* = 0.661, Wald *χ* ^2^ = 16.50, *P* = 0.680, and Wald *χ* ^2^ = 21.20, *P* = 0.116, resp.)

## 4. Discussion

In summary, we suggested that in asymptomatic patients with coronary artery disease, serum uric acid mildly relates to metabolic factor (type two diabetes mellitus, total cholesterol, and creatinine), bone-related proteins (osteopontin, osteoprotegerin), proinflammatory proteins (hs-C-reactive protein), left ventricular election fraction, and parameters that are attributed to vascular calcification (Agatston score index and coronary artery stenosis of more 50%). On the other hands, high quartile of serum uric acid associates well with Agatston score index, and after adjustment for type two diabetes mellitus, osteoprotegerin and osteopontin levels became a prognostic factor for coronary calcification. Recent clinical studies have shown that serum uric acid can correlate with vascular remodeling [23] and hemodynamic profiles [24] in several patient's populations. Because oxidative stress and inflammation are common in chronic heart failure beyond loss of left ventricular pump function [25, 26], we support a hypothesis that serum uric acid can contribute to influence of inflammatory activation, metabolic disorders, and calcium deposition in plaques and in vasculature [27]. Nevertheless, in the majority of studies there was a significant interrelationship between hyperuricemia (above 7 mg/dL) and cardiovascular outcomes [28–30]. Besides, recent clinical studies have shown that age and sex were significant effective modulators for the relation of serum uric acid to fatal congestive heart failure and ischemia events, with markedly stronger associations found in younger individuals [31, 32]. Although women and men present different age-related cardiovascular risk patterns, serum uric acid is independently and significantly associated with risk of cardiovascular events. Indeed, the Gubbio study showed a statistically significant contribution of serum uric acid to predict cardiovascular incidence in Italian population sample of 2469 men and women aged 35–74 years, free from major cardiovascular diseases, whereas the statistical contribution to predict coronary artery disease criteria were not significant [32]. Similar findings were true for elderly, postmenopausal women with established symptomatic coronary artery disease [33, 34], while investigators have emphasized that serum uric acid was not associated with mortality from acute, subacute, or chronic forms of coronary disease after adjustment for potential confounding cardiovascular risk factors. We obtained data about high predicted value of serum uric acid beyond obligatory hyperuricemia and vascular calcification in age- and sex-adjusted Cox proportional model. It has been postulated that gender-related cardiovascular disease incidences are associated with kidney function worsening, and serum uric acid elevation can reflect this interrelationship. No significant decrease of estimated glomerular filtration rate. plasma level was found in patients which were enrolled in the study. However, taking into consideration that serum uric acid would have a protective antioxidant activity [35, 36] we agreed that serum uric acid was an independent risk factor for coronary calcification, and it was able to preserve predict value in high concentration without several biological markers with defined potential toward vascular remodeling (osteopontin, osteoprotegerin, and type two diabetes mellitus). Theoretically, serum uric acid assessment may be useful in a population of children and adolescents with overweight or obese, as there is an association between the risk of asymptomatic coronary atherosclerosis and the level of uric acid in the blood [31]. Finally, we presumed that the dual paradoxical action of serum uric acid in the pathophysiology of asymptomatic coronary artery disease patients with preserved left ventricular systolic function required further discussions.

*In conclusion*, we suggested that serum uric acid level in the highest quartile (above 35.9 mmol/L) was very significant in prediction of coronary calcification examined by Agatston score index in comparison with the lowest quartile in asymptomatic coronary artery disease subjects with preserved left ventricular systolic function.

## 5. Limitations of the Study

This study has some limitations. We believed that a greater cohort would be desirable to improve the power of the study. We also relied on clinical data to rule out infection and other inflammatory diseases before sampling, but we cannot exclude that some patients had unrecognized conditions responsible for the elevated serum uric acid levels observed. We suppose to mean that these limitations might not have a significant influence on study data interpretation.

## Figures and Tables

**Table 1:** General characteristics of study patients.

	All patients (*n* = 126)
Age, years	58.34 ± 9.60
Male, *n* (%)	74 (58.7%)
Arterial hypertension, *n* (%)	84 (66.7%)
Hyperlipidaemia, *n* (%)	56 (44.4%)
2nd type diabetes mellitus, *n* (%)	46 (36.5%)
Premature CAD in family anamnesis, *n* (%)	12 (9.5%)
Smoking, *n* (%)	26 (20.6%)
eGFR, mL/min/m^2^	82.3 (95% CI = 58.7–102.6)
HbA1c, %	6.8 (95% CI = 4.1–9.5)
Fasting glucose, mmol/L	5.20 (95% CI = 3.3–9.7)
Creatinin, *μ*mol/L	86.74 (95% CI = 67.6–102.1)
Osteopontin, ng/mL	43.55 (95% CI = 31.5–57.0).
Osteoprotegerin, pg/mL	3849.51 (95% CI = 3282.23–4413.79).
hs-C-RP, mg/L	4.95 (95% CI = 3.15–9.80)
TC, mmol/L	5.1 (95% CI = 3.9–6.1)
LDL cholesterol, mmol/L	3.23 (95% CI = 3.11–4.4)
HDL cholesterol, mmol/L	0.91 (95% CI = 0.89–1.12)
Mean systolic BP, mm Hg	130.90 ± 8.41
Heart rate, beat per min	70.52 ± 3.34
LV EF, %	42.80 ± 0.76
HD-NCP	31 (95% CI = 21–56)
LD-NCP	25 (95% CI = 13–48)
CAP	96 (95% CI = 31–102)
Agatston' score index	586 (95% CI = 401–838)
Numerous coronary arteries with plaques determined	
1 vessel	46 (36.5%)
2 vessels	42 (33.3%)
3 vessels and more	38 (30.2%)
ACEI/ARBs	126 (100%)
Aspirin	98 (77.8%)
Other antiaggregants	6 (4.8%)
Statins	94 (74.6%)
Metformin	41 (32.5%)

CI: confidence interval, TC: total cholesterol, HbA1c: glycated hemoglobin, ACEI: angiotensin-converting enzyme inhibitor, ARBs: angiotensin-2 receptor blockers, HD-NCP: high-density noncalcified atherosclerotic plaque, LD-NCP: low-density noncalcified atherosclerotic plaque, and CAP: calcified atherosclerotic plaques.

**Table 2:** Tests of generalized regression model for six parameters (type two diabetes mellitus, total cholesterol, osteopontin, osteoprotegerin, high-sensitive C-reactive protein, and serum uric acid) that potentially contributed to calcification of coronary arteries.

Source	Dependent variables					
	% AS > 50%			Agatston' score index		
	*B*	Wald *χ* ^2^ test	Sig.	*B*	Wald *χ* ^2^ test	Sig.
T2DM	0.376	11.096	0.001	5.391	0.036	0.849
TC	0.144	3.221	0,073	33.45	2.759	0.097
OPN	0.01	0.001	0.974	1.206	1.957	0.162
OPG	0.21	17.698	0.000	0.265	99.271	0.000
hs-CRP	0.140	0.488	0.485	0.241	0.002	0.962
SUA	0.101	0.136	0.112	0.442	0.266	0.016

T2DM: type two diabetes mellitus, TC: total cholesterol, OPN: osteopontin, OPG: osteoprotegerin, hs-CRP: high-sensitive C-reactive protein, and SUA: serum uric acid.

**Table 3:** Tests of generalized regression model for five parameters (total cholesterol, osteopontin, osteoprotegerin, high-sensitive C-reactive protein, and serum uric acid) that potentially contributed to calcification of coronary arteries after excluding type two diabetes mellitus.

Source	Dependent variables					
	% AS > 50%			Agatston score index		
	*B*	Wald *χ* ^2^ test	Sig.	*B*	Wald *χ* ^2^ test	Sig.
TC	0.11	3.12	0,142	33.45	2.210	0.115
OPN	0.03	0.012	0.764	1.694	5.468	0.014
OPG	0.160	10.36	0.001	0.06	137.684	0.000
hs-CRP	0.132	1.32	0.615	0.363	0.006	0.941
SUA	0.021	0.120	0.680	0.442	88.27	0.016

TC: total cholesterol, OPN: osteopontin, OPG: osteoprotegerin, hs-CRP: high-sensitive C-reactive protein, and SUA: serum uric acid, and % AS: percent of coronary artery stenosis.

**Table 4:** Multivariable-adjusted odds ratios for vascular calcification examined by Agatston' score index by SUA quartiles. Odds ratios are adjusted for age, sex, numerous damaged coronary arteries, creatinin plasma level, fasting glucose, HbA1c, type two diabetes mellitus, and total cholesterol calculated versus low quartile.

SUA quartiles	SUA concentration, mmol/L		Odds ratios	95% CI	*P* value
	Mean value	95% CI			
Q1	12.25	11.31–13.19	1.00	—	—
Q2	17.33	16.45–18.22	1.01	0.82–1.16	0.68
Q3	25.29	23.53–27.05	1.03	0.76–1.20	0.59
Q4	39.27	35.05–43.48	1.46	1.22–1.98	0.001

Q: quartile, CI: confidence interval.
